# Neuregulin-1*β* Protects the Rat Diaphragm during Sepsis against Oxidative Stress and Inflammation by Activating the PI3K/Akt Pathway

**DOI:** 10.1155/2020/1720961

**Published:** 2020-07-19

**Authors:** Hua Liu, Xiao-jian Weng, Jun-yan Yao, Jun Zheng, Xiang Lv, Xu-hui Zhou, Hong Jiang, Shi-tong Li

**Affiliations:** ^1^Department of Anesthesiology and Critical Care Medicine, Shanghai Ninth People's Hospital, Shanghai Jiao Tong University School of Medicine, Shanghai 200011, China; ^2^Department of Anesthesiology and SICU, Xinhua Hospital, School of Medicine, Shanghai Jiao Tong University, Shanghai 200092, China; ^3^Department of Anesthesiology, Shanghai General Hospital, Shanghai Jiao Tong University School of Medicine, Shanghai 200080, China; ^4^Department of Pathology Center, Shanghai General Hospital, Shanghai Jiao Tong University School of Medicine, Shanghai 200080, China

## Abstract

Sepsis-induced diaphragm dysfunction (SIDD) which is mainly characterized by decrease in diaphragmatic contractility has been identified to cause great harms to patients. Therefore, there is an important and pressing need to find effective treatments for improving SIDD. In addition, acetylcholinesterase (AChE) activity is a vital property of the diaphragm, so we evaluated both diaphragmatic contractility and AChE activity. Though neuregulin-1*β* (NRG-1*β*) is known to exert organ-protective effects in some inflammatory diseases, little is known about the potential of NRG-1*β* therapy in the diaphragm during sepsis. Our study was aimed at exploring the effects of NRG-1*β* application on diaphragmatic contractility and AChE activity during sepsis. Proinflammatory cytokines, muscle injury biomarkers in serum, contractile force, AChE activity, proinflammatory cytokines, oxidative parameters, histological condition, terminal deoxynucleotidyl transferase-mediated dUTP nick end labeling (TUNEL) staining, and expression of phosphoinositide 3-kinase (PI3K)/protein kinase B (PKB/Akt) signaling proteins in the diaphragm were measured and compared between nonseptic and septic groups with or without NRG-1*β* treatment. In vitro, the effects of NRG-1*β* on reactive oxygen species (ROS) production in the lipopolysaccharide- (LPS-) stimulated L6 rat muscle skeletal cells with or without the Akt inhibitor MK-2206 were detected. NRG-1*β* inhibited proinflammatory cytokine release and muscle injury biomarkers soaring in serum and improved the sepsis-induced diaphragm dysfunction and AChE activity decrease significantly during sepsis. Meanwhile, the inflammatory response, oxidative stress, pathological impairment, and cell apoptosis in the diaphragm were mitigated by NRG-1*β*. And NRG-1*β* activated the PI3K/Akt signaling in the diaphragm of septic rats. Elevated ROS production in the LPS-stimulated L6 rat skeletal muscle cells was reduced after treatment with NRG-1*β*, while MK-2206 blocked these effects of NRG-1*β*. In conclusion, our findings underlined that NRG-1*β* could reduce circulating levels of proinflammatory cytokines in rats with sepsis, adjust diaphragmatic proinflammatory cytokine level, mitigate diaphragmatic oxidative injury, and lessen diaphragm cell apoptosis, thereby improving diaphragmatic function, and play a role in diaphragmatic protection by activating PI3K/Akt signaling.

## 1. Introduction

Sepsis is a series of clinical syndromes induced by severe infection, causing multiple organ dysfunctions [1]. It is often accompanied by acute respiratory failure, in which the weakness of respiratory muscles is an important factor [2]. Diaphragmatic dysfunction and weakness were identified during sepsis, leading to the specific definition of sepsis-induced diaphragm dysfunction (SIDD) [3, 4]. The diaphragm is the major respiratory muscle, and it is crucial for optimal respiration. SIDD can impair the ability of the respiratory pump, further leading to respiratory failure, weaning failure of mechanical ventilation, extended stay time in the intensive care unit (ICU), and even death [2, 5]. The common pathological events of diaphragmatic dysfunction during sepsis are complex, including inflammation, oxidative stress, metabolic imbalance, mitochondrial dysfunction, muscle apoptosis, and atrophy [6, 7]. The inflammation and oxidative stress are two main factors underlying diaphragmatic dysfunction during sepsis [8]. Inflammatory cytokine infiltration and recessive reactive oxygen species (ROS) generated from oxidative stress during sepsis could do damage to the skeletal muscle contractility-associated proteins such as sarco(endo)plasmic reticulum calcium-ATPases (SERCA) and/or sarcomere, subsequently causing diaphragmatic weakness [9]. In addition, acetylcholinesterase (AChE) activity at the neuromuscular junction (NMJ) is another vital property of the diaphragm. Clinically, muscle relaxant antagonists are often needed to help restore the muscle contractile tension of patients after general anaesthesia [10, 11]. Muscle relaxant antagonists exert their therapeutic effect by inhibiting AChE activity, which causes more acetylcholine release and ultimately accelerates muscle function restoration [12]. Our previous study verified that AChE activity decreased at the NMJ in the diaphragm during sepsis and oxidative stress was a vital contributor [13]. Drugs could inhibit the inflammation, and/or oxidative stress is theoretically useful for maintaining diaphragmatic contractile function and AChE activity during sepsis.

Neuregulin-1 (NRG-1) belongs to the neuregulin family, which was first discovered and studied in neural and cancer cells [14]. Both peripheral nerves and skeletal muscle are assumed to synthesize and excrete NRG-1. NRG-1 contains an epidermal growth factor- (EGF-) like domain that can bind to and activate receptor tyrosine kinases of the ErbB family (ErbB2, ErbB3, and ErbB4), exerting its role in cell survival, proliferation, migration, and differentiation [15, 16]. All of the ErbB receptors are mainly present at the NMJ [17], and NRG-1 acts as a medium between terminal Schwann cells and motor axons, between motor axons and muscles, and between different muscle fibres [18, 19]. Previous studies have shown that sepsis can induce a reduction in NRG-1 in the skeletal muscle system [20, 21]. Both in vivo and in vitro studies have demonstrated that NRG-1 and its associated signaling pathway are protective against injury induced by inflammatory diseases in the cardiovascular and nervous systems and simultaneously improve organ function [22–25]. However, the effects of NRG-1*β* on diaphragmatic contractility and AChE activity during sepsis have yet been explored. These findings provide a theoretical basis for relieving diaphragmatic dysfunction by exogenously administering NRG-1*β*. Our present study was aimed at exploring the role of NRG-1*β* on diaphragmatic contractility and AChE activity and simultaneously analyzing its effects on proinflammatory cytokines (tumor necrosis factor-*α* (TNF-*α*), interleukin-1*β* (IL-1*β*), and IL-6) in serum and the diaphragm, oxidative parameters (thiobarbituric acid reactive species (TBARS), protein carbonyl contents, and myeloperoxidase (MPO) activity) in the diaphragm, nonspecific muscle injury parameters (aspartate transaminase (AST), lactate dehydrogenase (LDH), and creatine kinase (CK) activity and myohemoglobin (Myo) content) in serum, and specific diaphragm injury (hematoxylin-eosin (H&E) staining of diaphragm) and diaphragm cell apoptosis (terminal deoxynucleotidyl transferase-mediated dUTP nick end labeling (TUNEL) staining of the diaphragm) during sepsis. And we detected the phosphoinositide 3-kinase (PI3K)/protein kinase B (PKB/Akt) signaling proteins in the diaphragm using Western blotting. We further detected the effects of NRG-1*β* on reactive oxygen species (ROS) production in the lipopolysaccharide- (LPS-) stimulated L6 rat skeletal muscle cells with or without the Akt inhibitor MK-2206 in order to determine whether NRG-1*β* could protect rat skeletal muscle cells against oxidative stress during sepsis and whether this effect could be suppressed by the Akt inhibitor. Here, we provide evidence that NRG-1*β* was a therapeutic strategy for SIDD against oxidative stress and inflammation by activating the PI3K/Akt pathway.

## 2. Materials and Methods

### 2.1. Animals and Experimental Design

Our study was approved by the Animal Care and Use Committee of Shanghai General Hospital Affiliated with Shanghai Jiao Tong University (grant number 2017DW001). The experiment was performed using a total of 28 Sprague-Dawley rats weighing 220-260 g. All animals were allowed free access to food and water and were housed at a temperature of 24 ± 1°C under a 12 h light-dark cycle (lights on from 8 am to 8pm).

The rats were randomized into 3 groups: the sham group (*n* = 6), the sepsis group (*n* = 11), and the NRG group (*n* = 11). Sepsis was induced by caecal ligation and puncture (CLP) which was performed as described in our previous study [13, 26]. In brief, a 3 cm midline abdominal incision was made under aseptic conditions after the rats were anaesthetized with an intraperitoneal injection of pentobarbital (50 mg/kg). The caecum was ligated at a distance equal to 50% of the total length of the caecum below the ileocaecal valve and perforated once in the mesenteric-to-antimesenteric direction with an 18-gauge needle. Then, the caecum was pressed gently to produce droplets of faeces from the 2 puncture sites. Finally, the caecum was replaced into the peritoneal cavity, and the abdominal wall was closed in 2 layers with 3-0 silk sutures. The rats in the sham group underwent a similar procedure, but the caecum was neither ligated nor perforated. Shortly after the surgery, all rats received 10 ml prewarmed (37°C) normal saline subcutaneously.

Thirty minutes before surgery, the rats in the NRG group received tail vein injections of NRG-1*β* (10 *μ*g/kg, PeproTech, Rocky Hill, NJ, USA), whereas the rats in the other two groups received an equal volume of normal saline.

Twenty-four hours after surgery, all surviving rats were euthanized by an intraperitoneal injection of pentobarbital (100 mg/kg), and then blood samples were collected from the inferior vena cava, centrifuged, and stored at -80°C until the assay. Then, the diaphragms were harvested for subsequent tests. A muscle strip (approximately 5 mm wide) was obtained from the midcoastal region of the left diaphragm to immediately measure the diaphragmatic contractile force. The remaining part of the left diaphragm was immediately stored at -80°C until it was assayed for Western blotting of p-PI3K, pan-PI3K, p-Akt, pan-Akt, and actin. The coastal region of the right hemidiaphragm was immediately removed for histological examination and TUNEL staining. Meanwhile, the remaining part was immediately stored at -80°C until it was assayed for proinflammatory cytokines, AChE activity, TBARS content, protein carbonyl content, and MPO activity.

### 2.2. Diaphragmatic Contractile Property Measurement

Diaphragmatic contractile properties were measured according to previous studies [27, 28]. The diaphragm strip was suspended vertically in a 37°C tissue bath filled with Krebs solution containing 137 mM NaCl, 4 mM KCl, 2 mM CaCl_2_, 1 mM MgCl_2_, 1 mM KH_2_PO_3_, 12 mM NaHCO_3_, and 6.5 mM glucose (pH 7.40 ± 0.05). Additionally, 12 *μ*M d-tubocurarine (Sigma-Aldrich, St. Louis, MO, USA) was added to the Krebs solution to prevent neuromuscular transmission, and the solution was bubbled with 95% O_2_/5% CO_2_. Two silver stimulating electrodes were placed parallel to the muscle strip and connected to an electrical stimulator (ALC-MPA2000-S; Alcott Biotech, Shanghai, China) that could be activated by a computer. After a 15 min thermoequilibration period, the optimal length (*L*_0_) at which the muscle generated peak twitch force was determined. The stimulus intensity was then set to 120% of the voltage intensity, which was adjusted when the maximal twitch response was reached at the *L*_0_ to ensure supramaximal stimulation. Each strip was stimulated with an ms train every 2 min at the following frequencies: 10, 20, 40, 60, 80, and 120 Hz.

Following the experiment, the muscle strip was blotted dry and weighed. Finally, the force was normalized to the muscle cross-sectional area (CSA), which was calculated by the following formula (assuming that muscle density was 1.056 g/cm^3^): CSA = muscle weight (g)/[*L*_0_ (cm) × 1.056 (g/cm^3^)].

### 2.3. Biochemical Measurement of AChE Activity in the Diaphragm

The AChE activity was measured according to our previous research [13]. In brief, the AChE activity measurement was conducted using an AChE assay kit from the Nanjing Jiancheng Bioengineering Institute (A024, Nanjing, China) according to the manufacturer's instructions.

### 2.4. Enzyme-Linked Immunosorbent Assays for TNF-*α*, IL-1*β*, and IL-6 in Serum and the Diaphragm

The serum and diaphragmatic levels of TNF-*α*, IL-1*β*, and IL-6 were measured using enzyme-linked immunosorbent assay (ELISA) kits (Elabscience Biotechnology Co., Ltd., Wuhan, China) according to the manufacturer's instructions.

### 2.5. Measurement of AST, LDH, and CK Activity and Myo Content in Serum

#### 2.5.1. Measurement of AST Activity

AST activity was measured using an Aspartate Aminotransferase Activity Assay kit (105135, Abcam, US) according to the manufacturer's instructions. Briefly, AST catalyses the reaction as follows:

(1) $$\begin{array}{c} \text{aspartate}+\alpha ‐\text{ketoglutarate}=\text{oxaloacetate}+\text{glutamate} \end{array}$$

Glutamate was detected by the conversion of a nearly colourless probe to a coloured product that was detected at 450 nm. In detail, after freezing-thawing the serum samples, prepare test samples, standard, and positive control of up to 50 *μ*l/well with assay buffer in a 96-well plate. Then, add 100 *μ*l of the reaction mix to each well. After reaction, glutamate production was measured according to the glutamate standard curve and the corresponding optical density. One unit of AST was defined as the amount of AST required to generate 1.0 *μ*mol of glutamate per minute at 37°C.

#### 2.5.2. Measurement of LDH Activity

LDH activity was measured using a Lactate Dehydrogenase Assay kit (197000, Abcam, US) according to the manufacturer's instructions. LDH is a ubiquitous enzyme in vertebrate organisms that catalyses the reversible conversion of pyruvate to lactate and the concomitant conversion of NADH and NAD+. NADH converted a colourless probe to a coloured one. Then, the fluorescence (Ex/Em = 535/587 nm) was measured in a kinetic mode at 37°C for 10-30 min. In detail, after freezing-thawing the serum samples, set up a plate for diluted standard (50 *μ*l), samples (50 *μ*l), and positive control wells (50 *μ*l). Then, add 50 *μ*l of reaction mix to each well. Finally, LDH activity in the samples was expressed as U/l of sample. One unit of LDH was defined as the amount of enzyme required to generate 1.0 *μ*mol of NADH per min at pH 8.8 and 37°C.

#### 2.5.3. Measurement of CK Activity

CK activity was measured using a Creatine Kinase Assay kit (k777-100, Biovision, USA) according to the manufacturer's instructions. CK converts creatine into phosphocreatine and ADP. The generated phosphocreatine and ADP reacted with the CK Enzyme Mix to form an intermediate that converted a colourless probe to a coloured product with a strong absorbance at 450 nm. In detail, after freezing-thawing the serum samples, prepare test samples, standard, and positive control of up to 50 *μ*l/well with assay buffer in a 96-well plate. Then, add 50 *μ*l of reaction mix to each well. Finally, CK activity in the samples was expressed as U/l of sample. One unit of CK was defined as the amount of enzyme required to generate 1.0 *μ*mol of NADH per min at pH 9.0 and 37°C.

#### 2.5.4. Measurement of Myo Content

Myo content was measured using the Myoglobin Rat ELISA kit (157739, Abcam, US) according to the manufacturer's instructions. Finally, the quantity of Myo in the serum was interpolated from the standard curve plotted from the standards and corrected for sample dilution.

### 2.6. Measurement of TBARS Contents in the Diaphragm

The formation of TBARS was determined by an acid-heating reaction, as previously described [29]. Specifically, prepare the homogenate after adding 100 *μ*l H_2_O_2_ to 30 mg diaphragm tissue. Vortex and centrifuge the homogenate after adding 150 *μ*l perchloric acid. Then, prepare the TBARS-TBA complex using the supernatant. Finally, add 200 *μ*l TBARS-TBA complex to each well for the test. After the reaction, TBARS levels were finally determined based on malondialdehyde (MDA) equivalents per milligram of protein by measuring the absorbance at 535 nm.

### 2.7. Measurement of Protein Carbonyls in the Diaphragm

The levels of protein carbonyls were measured based on the reaction with dinitrophenyl hydrazine, as previously described [30]. Specifically, vortex and centrifuge the 10% diaphragm homogenate first. Then, drain the supernatant and dilute it at the ratio of 1 : 9 for further test. After the reaction, the levels of PC were determined as nmol of protein carbonyls per milligram of protein by reading the absorbance at 375 nm.

### 2.8. Assay of MPO Activity in the Diaphragm

MPO activity was determined by using a test kit from Nanjing Jiancheng Bioengineering Institute (A044, Nanjing, China). The activity was determined as units of MPO activity per gram of tissue wet weight by spectrophotometrically measuring the change in absorbance at 412 nm.

### 2.9. Histopathological Procedure and Semiquantitative Analysis of Diaphragm Damage Score (DDS)

The harvested right hemidiaphragms were fixed in 4% formalin. After fixation, each tissue sample was routinely processed and embedded in paraffin. Then, 5 *μ*m thick longitudinal sections were produced from the tissue blocks and stained with hematoxylin and eosin. The sections were dehydrated in a series of graded ethanol solutions and sealed with neutral resin. Then, these sections were photographed with a digital camera (Axioplan 2, Carl Zeiss, Oberkochen, Germany) for histopathological examination. The degree and severity of the diaphragm injury were assessed and graded according to the 5-point semiquantitative scale consisting of edema, neutrophil infiltration, haemorrhages, distribution of myofibrils, etc., described in the previous study [31] by a professional pathologist who was blinded to the experimental groups. A severity grade was finally expressed as the diaphragm damage score (DDS).

### 2.10. TUNEL Staining of Diaphragm Cells and Quantitative Analysis of Cell Apoptosis Percentages from TUNEL-Stained Images

TUNEL staining was performed using the Apoptosis Detection Kit (C1088, Beyotime, China) according to the manufacturer's instructions. Finally, the images were captured using the Axioplan 2 imaging system and software; then, the TUNEL-positive myonuclei were quantified by Image-Pro Plus software (six images in three rats were analyzed) and indicated as a percentage of all myonuclei.

### 2.11. Cell Culture and Treatment and Measurement of ROS Production in the L6 Skeletal Muscle Cells

L6 rat skeletal muscle cells were purchased from Shanghai Institutes for Biological Science (Shanghai, China) and regularly cultured and differentiated according to our previous study [32]. Thirty minutes before LPS (Sigma-Aldrich) treatment, cells were treated with PBS (control group (C group) and LPS group (L group)), NRG-1*β* (10 nM, PeproTech, PBS+NRG group (CN group) and LPS+NRG group (LN group)), or NRG-1*β* plus the Akt inhibitor MK-2206 (10 *μ*M, Selleck, LPS+NRG+MK-2206 group (LNM group)) [33]. Then, except C and CN groups, cells of other groups were treated with LPS (1 *μ*g/ml) [32] for 24 h. At the end of incubation, cells were collected for quantifying intracellular ROS with a Reactive Oxygen Species Assay Kit (S0033, Beyotime, China) according to the manufacturer's instructions. Briefly, cells were resuspended with a serum-free medium containing the fluorescent probe DCFH-DA (10 *μ*M). Subsequently, cells were incubated at 37°C for 20 min in the dark. DCF fluorescence intensity was measured with a microplate absorbance reader.

### 2.12. Western Blotting Analysis

Firstly, the protein concentration of the diaphragm sample was determined using a bicinchoninic acid protein assay kit (Beyotime, Shanghai, China) after being homogenized in radioimmunoprecipitation lysis buffer (Beyotime) containing 1 mM phenylmethanesulfonyl fluoride (Amresco, LLC, Solon, OH, USA). Subsequently, 30 *μ*g of the proteins was loaded on a 10% sodium dodecyl sulfate- (SDS-) polyacrylamide gel for electrophoresis. Then, the proteins were transferred to polyvinylidene difluoride membranes (Millipore, Billerica, MA, USA) which were blocked in Western blocking buffer (Beyotime) for 1.5 h at room temperature and incubated with primary antibodies at 4°C overnight for immunoblotting. The primary antibodies used were rabbit antibodies specific for p-PI3K (Tyr607) (1 : 1000; ab182651; Abcam), pan-PI3K (1 : 500; BS3678; Bioworld Technology, Nanjing, Jiangsu, China), p-Akt (Thr308) (1 : 1000; #2965; Cell Signaling Technology (CST), Boston, MA, USA), and pan-Akt (1 : 1000; #4691, CST) and a mouse antibody specific for actin (1 : 1000; AF0006; Beyotime). The blots were incubated for 1 h at room temperature with peroxidase-conjugated secondary antibodies after rinsing three times in Tris-buffered saline with Tween 20 (TBST). Finally, the membranes were washed with TBST and visualized using an electrochemiluminescence detection kit (Millipore). Densitometry was conducted using Image-Pro Plus software (version 6.0; Media Cybernetics, Inc.).

### 2.13. Statistical Analyses

Statistical analyses were conducted using SPSS (version 19.0; SPSS Inc., Chicago, Illinois, USA). The values are presented as the mean ± standard deviation (SD). The data were first tested for normality and equality of variance. Between-group comparisons for each dependent variable were assessed by analysis of variance (ANOVA) with the least significant difference (LSD) test. *P* < 0.05 was considered to be statistically significant.

## 3. Results

### 3.1. Survival Rates within 24 h

Six of the 11 rats in the sepsis group and 9 of the 11 rats in the NRG group survived to 24 h after the CLP procedure, whereas all of the rats in the sham group survived. Additionally, the surviving rats that underwent CLP surgery developed varying degrees of septic symptoms, such as a loss of movement, hair erection, shortness of breath, subconjunctival exudation, and diarrhoea.

### 3.2. Diaphragm Force-Frequency Relationship

**Figure 1:**
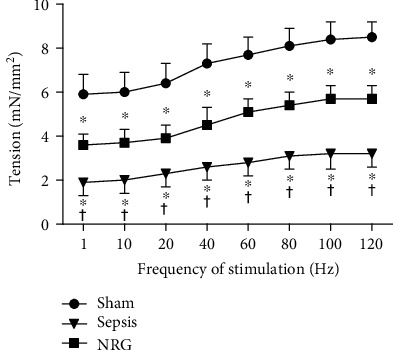
Force-frequency curves of the diaphragm strips. The data are presented as the mean ± SD. ^∗^*P* < 0.01 vs. the sham group and ^†^*P* < 0.01 vs. the sepsis group.

The data of the response of the diaphragm strips to stimuli of increasing frequencies are shown in Figure 1. A significant downward shift across all of the applied stimulation frequencies (10-120 Hz) was observed in the sepsis group (*P* < 0.01). NRG-1*β* treatment partially restored the downward shift (*P* < 0.01), which suggested that NRG-1*β* can improve the reduced diaphragmatic force generation during sepsis.

### 3.3. AChE Activity in the Diaphragm

**Figure 2:**
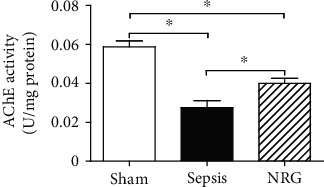
AChE activity in the three groups of the rat diaphragm. The data are presented as the mean ± SD. ^∗^*P* < 0.01.

Compared with the sham group, AChE activity decreased significantly in both the sepsis and the NRG groups 24 h after surgery (*P* < 0.01). However, AChE activity increased in the NRG group compared with the sepsis group (*P* < 0.01) (Figure 2).

### 3.4. Effects of NRG-1*β* on Serum and Diaphragmatic Proinflammatory Cytokines

**Figure 3:**
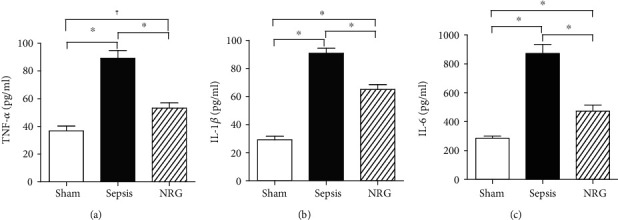
Levels of TNF-*α*, IL-1*β*, and IL-6 in the three groups of rat serum. The data are presented as the mean ± SD. ^∗^*P* < 0.01, ^†^*P* < 0.05.

As shown in Figure 3, the levels of serum TNF-*α*, IL-1*β*, and IL-6 were increased significantly in rats in the sepsis group compared to rats in the sham group 24 h after surgery (*P* < 0.01), while TNF-*α*, IL-1*β*, and IL-6 concentrations at 24 h after CLP were reduced after NRG-1*β* treatment (*P* < 0.01).

**Figure 4:**
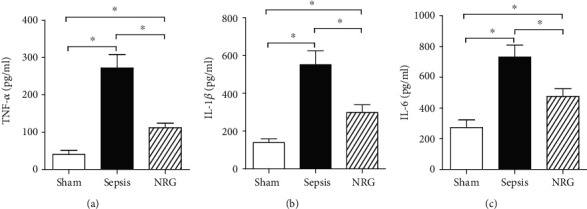
Levels of TNF-*α*, IL-1*β*, and IL-6 in the three groups of the rat diaphragm. The data are presented as the mean ± SD. ^∗^*P* < 0.01.

As shown in Figure 4, the levels of diaphragmatic TNF-*α*, IL-1*β*, and IL-6 were increased significantly in rats in the sepsis group compared to rats in the sham group 24 h after surgery (*P* < 0.01), while TNF-*α*, IL-1*β*, and IL-6 concentrations at 24 h after CLP were reduced after NRG-1*β* treatment (*P* < 0.01).

### 3.5. Effects of NRG-1*β* on Serum Biomarkers of Muscle Injury

**Figure 5:**
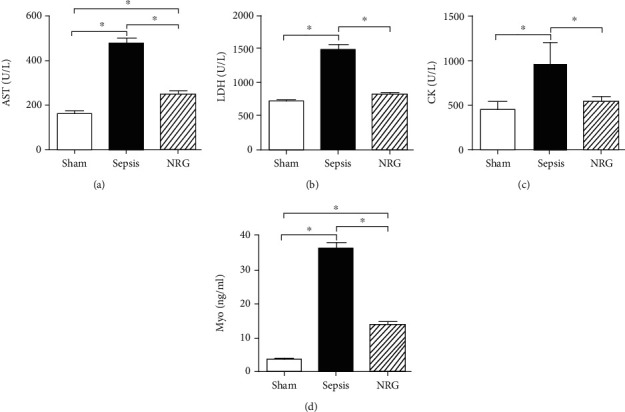
AST, LDH, and CK activity and Myo content in the three groups of the rat diaphragm. The data are presented as the mean ± SD. ^∗^*P* < 0.01.

As there is no specific biomarker for skeletal muscle injury, we detected the AST, LDH, and CK activity and the Myo content in serum to evaluate muscle injury generally. The AST, LDH, and CK activity and the Myo content in the serum rose significantly 24 h after CLP (*P* < 0.01), while the levels dropped significantly upon NRG-1*β* treatment (*P* < 0.01) (Figure 5).

### 3.6. Effects of NRG-1*β* on Oxidative Injury of the Diaphragm

**Figure 6:**
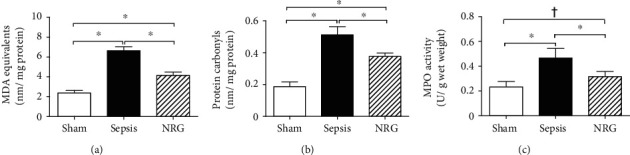
TBARS, protein carbonyl content, and MPO activity in the three groups of the rat diaphragm. The data are presented as the mean ± SD. ^∗^*P* < 0.01, ^†^*P* < 0.05.

As shown in Figure 6, the TBARS, protein carbonyl contents, and MPO activity in the diaphragm were increased significantly in rats in the sepsis group compared to rats in the sham group 24 h after surgery (*P* < 0.01), while the increase was partially restored after NRG-1*β* treatment (*P* < 0.01, *P* < 0.05).

### 3.7. Effects of NRG-1*β* on Histological Conditions in the Diaphragm

**Figure 7:**
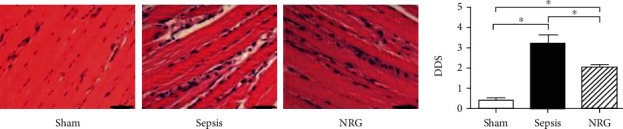
Histological conditions and DDS in the three groups of the rat diaphragm. The data are presented as the mean ± SD. ^∗^*P* < 0.01. Bars = 50 *μ*m.

As shown in Figure 7, histological examinations of the diaphragm were conducted in the three groups. H&E staining of diaphragms from the rats in the sham group revealed a normal structure of the diaphragm (polygonal pink fibres with several blue/purple cellular membranes and a neat and tight arrangement of muscle fibres). Twenty-four hours after the CLP procedure, obvious changes were noted in the diaphragms of the rats in the sepsis group; these diaphragms presented a disordered arrangement, more inflammatory cell infiltration, and widened intercellular spaces. The pathological changes were partially improved by NRG-1*β* treatment. The diaphragm fibre arrangement was more regular; intercellular edema and haemorrhages were alleviated. Additionally, the diaphragms of the rats in the NRG group exhibited lesser inflammatory infiltration. The semiquantitative analysis showed that the DDS was higher in the sepsis group than in the sham group, while NRG-1*β* treatment decreased the DDS significantly (*P* < 0.01).

### 3.8. Effects of NRG-1*β* on Cell Apoptosis of the Diaphragm

**Figure 8:**
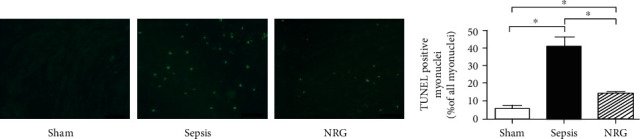
TUNEL staining and cell apoptosis percentages from TUNEL-stained images in the three groups of the rat diaphragm. The data are presented as the mean ± SD. ^∗^*P* < 0.01. Bars = 50 *μ*m.

As shown in Figure 8, TUNEL staining was conducted to measure the apoptotic cells in the diaphragm. The TUNEL-positive myonuclei increased in the sepsis group compared with the sham group (*P* < 0.01). However, the TUNEL-positive myonuclei decreased significantly in the NRG group compared with the sepsis group (*P* < 0.01) although the TUNEL-positive myonuclei in the NRG group were still higher than those in the sham group (*P* < 0.01).

### 3.9. Effects of NRG-1*β* on the PI3K-Akt Signaling Pathway in the Diaphragm

**Figure 9:**
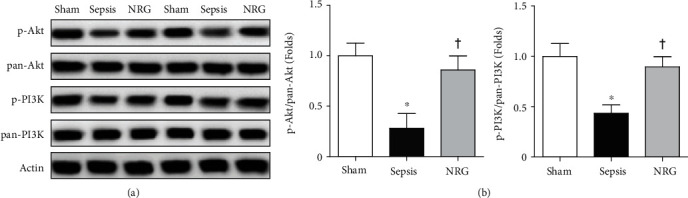
(a) Western blotting analyses of p-PI3K and p-Akt expressions in the three groups of the rat diaphragm. Representative blots from 2 of the 6 subjects in each group are displayed. (b) The corresponding bar graphs. Actin and pan-PI3K and Akt expressions serve as the internal control. Data are presented as the mean ± SD. ^∗^*P* < 0.01 vs. the sham group; ^†^*P* < 0.05 vs. the sepsis group.

As shown in Figure 9, the expressions of p-PI3K and p-Akt were reduced during sepsis (*P* < 0.01), while the expression of these proteins increased after NRG-1*β* treatment (*P* < 0.05).

### 3.10. Effects of NRG-1*β* on ROS Production in the L6 Rat Skeletal Muscle Cells

**Figure 10:**
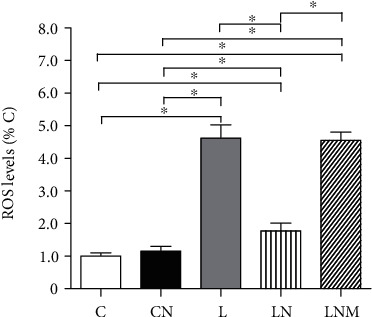
ROS levels in the L6 skeletal muscle cells of different groups (presented as percentages of the C group). Data are presented as the mean ± SD. ^∗^*P* < 0.01. C: control group; CN: PBS+NRG group; L: LPS group; LN: LPS+NRG group; LNM: LPS+NRG+MK-2206 group.

As shown in Figure 10, ROS production in the L group increased significantly compared with that in the C group and CN group (*P* < 0.01), while there is no significant difference in ROS production between the C group and CN group. Treatment with NRG-1*β* decreased the ROS production (*P* < 0.01), while the administration of MK-2206 blocked these effects of NRG-1*β* (*P* < 0.01).

## 4. Discussion

In the current study, we observed the effects of the NRG-1*β* on the diaphragm in a rat model of sepsis. Our study indicated that NRG-1*β* exerted diaphragm-protective functions, i.e., strengthening diaphragmatic contractile force and AChE activity, alleviating inflammatory infiltration, reducing oxidative and morphological injury, and lessening diaphragm cell apoptosis. Moreover, NRG-1*β* activated the PI3K/Akt signaling pathway in the diaphragm during sepsis. And NRG-1*β* protected LPS-stimulated L6 skeletal muscle cells against oxidative stress by activating the PI3K/Akt pathway, which was further confirmed.

These observations should be of clinical relevance, since SIDD causes great harms as mentioned before, which finally leads to a heavy social and financial burden. We firstly offered the evidence that NRG-1*β* may provide a therapeutic option for curing SIDD via suppressing oxidative stress and inflammation by activating the PI3K/Akt signaling pathway.

In this study, sepsis was established by CLP, as described in a previous study [26]. CLP is regarded as the gold standard model of sepsis [34]. Twenty-four hours after CLP, there were a significant increase in proinflammatory cytokines (TNF-*α*, IL-1*β*, and IL-6) in serum and an elevated death rate in rats, while the surviving rats developed septic syndrome; this demonstrated that our sepsis model was successfully established.

NRG-1 is a member of the neuregulin family, and it has two isoforms, namely, NRG-1*α* and NRG-1*β*, with different EGF-like domains. NRG-1*β* is 10-100 times more potent than NRG-1*α* [15, 16]; thus, we chose to use NRG-1*β* in this study. Proinflammatory cytokines such as TNF-*α*, IL-1*β*, and IL-6 have been verified to participate in and promote sepsis in previous studies; similarly, our study suggested that sepsis-induced diaphragmatic dysfunction and AChE activity decrease were associated with an increased exposure of the diaphragm to proinflammatory cytokines. NRG-1*β*'s anti-inflammatory roles on brains have been noted previously [22], but little is known whether it could also protect the diaphragm during sepsis via its anti-inflammatory effects. In this study, we clearly demonstrated that NRG-1*β* treatment substantially decreased the systemic and diaphragmatic proinflammatory cytokines. Meanwhile, inflammatory infiltration in the diaphragm during sepsis was also ameliorated after NRG-1*β* treatment.

Additionally, oxidative stress which is characterized by the excessive production of ROS [8, 35] has been proven to play an essential role in the onset and development of sepsis. ROS consist of active oxygen free radicals or molecules such as superoxide (O_2_^∙−^), hydrogen peroxide (H_2_O_2_), hydroxyl radicals (OH), and hypochlorous acid (HOCl) [36, 37], which can attack proteins, unsaturated lipids, nucleic acids, and carbohydrates, subsequently triggering injury in tissues and organisms. Malondialdehyde (MDA, detected by TBARS) and protein carbonyls are two typical markers of oxidative stress-induced injury to lipids and proteins, respectively [29, 30]. We measured TBARS and protein carbonyl levels to observe oxidative injury in the diaphragm of rats; simultaneously, we tested serum AST, LDH, and CK activity and Myo content to observe skeletal muscle injury indirectly, as in previous studies [38, 39]. Results from the current study showed that sepsis induced severe oxidative injury in the diaphragm. Fortunately, NRG-1*β* treatment significantly reduced the serum AST, LDH, and CK activity and Myo content during sepsis. Moreover, NRG-1*β* administration could reduce TBARS and protein carbonyl levels in the diaphragm of rats with sepsis, which illustrated that sepsis-induced oxidative injury in the diaphragm was alleviated. These results were in line with its protective role against oxidative stress in the myocardium and neurons [22, 25]. Besides, AChE activity elevated after NRG-1*β* treatment as well, since our previous research suggested that oxidative stress played a vital role in AChE activity decrease in the diaphragm of rats with sepsis.

Interestingly, our study found that MPO activity of the diaphragm in the rats of the NRG group was significantly reduced compared to that of the sepsis group. MPO is synthesized and secreted by neutrophils and is regarded as a marker of inflammation and neutrophil infiltration in tissues [40]. Meanwhile, MPO can convert H_2_O_2_ into HOCl, which is a more active ROS than H_2_O_2_ [41]. Neutrophil infiltration, larger nuclei, and wider spacing of skeletal muscle fibres were observed in the diaphragms of rats with sepsis. After NRG-1*β* treatment, neutrophil infiltration was improved, which is consistent with the change in MPO activity. In addition, nuclear size, the spacing of skeletal muscle fibres, and DDS were diminished. These findings implied an interplay relationship of oxidative stress and inflammation in the development of SIDD while NRG-1*β* exerted its protective effects through alleviating both inflammatory and oxidative injuries in the diaphragm.

Our study showed that NRG-1*β* improved the inhibition of PI3K and Akt in the diaphragm of rats with sepsis, which is further verified in vitro since the protective effects of NRG-1*β* in the LPS-stimulated L6 skeletal muscle cells were blocked by the Akt inhibitor. Several previous studies have proven that NRG-1*β* could activate the PI3K/Akt signaling pathway and exert protective effects in cardiac myocytes subjected to a variety of pathological conditions [42, 43]. However, to the best of our knowledge, this study is the first to identify NRG-1*β* as an activator of a PI3K/Akt-mediated protective role against oxidative stress and inflammation in the diaphragm during sepsis. Both ErbB2 and ErbB3 receptors are verified to be expressed in the rat diaphragm [44]. NRG-1 could recruit and activate the PI3K through its EGF-like domain binding to the ErbB2/ErbB3 heterodimer [45]. Further studies are needed to detect the activation of the two ErbB receptors. The PI3K/Akt pathway is an important signal transduction pathway. Akt is the downstream target of PI3K and plays a pivotal role in several cellular processes, including cell proliferation, differentiation, apoptosis, migration, and transcription [46]. Akt inhibition is associated with an inflammatory response, while Akt activation reduces cellular oxidative stress and inhibits the release of proinflammatory cytokines [47, 48]. Several previous studies have reported that some drugs could prevent liver injury [49] or hepatic fibrosis [50] via regulating the PI3K/Akt pathway. These provide the theoretical foundation for our conclusions since we have proven that NRG-1*β* can activate the PI3K/Akt pathway in the diaphragm of rats with sepsis.

## 5. Conclusions

We consider that there were at least two highlights in the current study. First, our study found that NRG-1*β* could prevent diaphragmatic function and AChE activity during sepsis for the first time. Additionally, NRG-1*β* treatment also ameliorated the inflammatory infiltration, oxidative injury, and cell apoptosis in the diaphragm of the rats with sepsis. Second, the study showed that the PI3K/Akt pathway was responsible for NRG-1*β*'s protective roles on the diaphragm against inflammation and oxidative stress during sepsis. In conclusion, the current study confirmed NRG-1*β*'s diaphragm-protective role during sepsis and its related mechanisms, which would provide a new therapeutic vision of protecting SIDD in the future.

## Data Availability

The Tiff. data used to support the findings of this study are included within the article.
